# Polyunsaturated aldehydes induce distinct proteomic responses in two diatom-associated bacterial communities

**DOI:** 10.3389/fmicb.2026.1838042

**Published:** 2026-06-10

**Authors:** María Hernanz-Torrijos, María J. Ortega, Almudena Escobar-Niño, Francisco Javier Fernández-Acero, Ana Bartual

**Affiliations:** 1 University Marine Research Institute (INMAR) CEI-MAR International Campus of Excellence in Marine Science, University of Cádiz, Cádiz, Spain; 2 Department of Biology, Faculty of Marine and Environmental Science, University of Cádiz, Cádiz, Spain; 3 Department of Organic Chemistry-INBIO, Faculty of Marine and Environmental Sciences, University of Cádiz, Cádiz, Spain; 4 Microbiology and Proteomics Laboratory, Viticulture and Agri-food Research Institute (IVAGRO), University of Cádiz, Cádiz, Spain; 5 Department of Biomedicine, Biotechnology and Public Health, Faculty of Marine and Environmental Sciences, University of Cádiz, Cádiz, Spain

**Keywords:** diatom-bacteria interactions, microbial ecology, phycosphere, polyunsaturated aldehydes, proteomics

## Abstract

Diatom-derived polyunsaturated aldehydes (PUAs) significantly influence marine bacterial dynamics, yet the underlying proteomic mechanisms remain elusive. We employed high-resolution comparative proteomics to decipher the functional reprogramming of two bacterial communities—one naturally associated with a PUA-producing diatom (N-community) and another with a non-PUA producing (I-community)—under ecologically relevant PUA exposure. While growth rates and cell densities remained unaffected, indicating an absence of acute toxicity, proteomics revealed pronounced community-specific reorganization. N-community displayed stable, regulation-oriented adjustments consistent with physiological accommodation, whereas I-community exhibited dose-dependent stress responses at the endpoint of the growth curve, shifting toward protein repair and antioxidant defense. Our findings demonstrate that PUAs trigger profound proteomic reprogramming conditioned by the communities’ prior ecological history. This functional divergence provides a molecular basis for understanding bacterial fitness and succession during diatom blooms, where PUA-mediated interactions could act as a selective filter shaping the phycosphere’s microbial landscape.

## 1 Introduction

Relationships between diatoms and bacteria constitute one of the most relevant interactions in marine ecosystems, due to their ubiquity and functional impact on the global carbon cycle, nutrient regeneration and the productivity and stability of aquatic food web (Cole, 1982; Azam and Malfatti, 2007; Amin et al., 2012). Far from being passive associations, these relationships involve a bidirectional exchange of organic and inorganic compounds, along with chemical signals that modulate the physiological activity, behavior, and community composition of both partners (Amin et al., 2015; Durham et al., 2015; Cirri and Pohnert, 2019). These processes occur within the immediate microenvironment surrounding phytoplankton cells, the phycosphere (Bell and Mitchell, 1972). Enriched in organic compounds exuded by the cell, the phycosphere is recognized as a chemically dynamic and ecologically significant microscale hotspot (Seymour et al., 2017). Among the many chemical mediators proposed to influence phytoplankton–bacteria interactions within the phycosphere, polyunsaturated aldehydes (PUA) have received increasing attention (Balestra et al., 2011; Pepi et al., 2017; Eastabrook et al., 2020; Hernanz-Torrijos et al., 2023). PUA are long-chain oxylipins derived from the lipoxidation of intracellular polyunsaturated fatty acids (Fontana et al., 2007), produced and released into the environment by different phytoplankton groups, particularly diatoms (Miralto et al., 1999).

Current evidence indicates that PUA can induce diverse and sometimes contrasting effects on marine bacteria. Some studies have reported that PUA do not produce significant effects on bacterial abundance or community composition following exposure (Paul et al., 2012; Eastabrook et al., 2020), whereas others describe responses ranging from bacteriostatic effects and growth inhibition to tolerance or even stimulation, depending on PUA concentration and the bacterial taxa or strain involved (Ribalet et al., 2008, 2014; Balestra et al., 2011; Pepi et al., 2017; Wu et al., 2021). Field-based experiments further demonstrate that PUA and other oxylipins dose-dependently regulate microbial activity on sinking particles, stimulating remineralization at moderate concentrations and inhibiting it at higher levels (Edwards et al., 2015). Importantly, these responses did not necessarily translate into increased bacterial growth, suggesting that oxylipin exposure may alter microbial functional states rather than implying stimulation or toxicity. Most of these studies have focused primarily on growth or community-level responses, indicating that PUA impacts depend strongly on concentration (Ribalet et al., 2008; Edwards et al., 2015), bacterial lifestyle (Wu et al., 2021), and/or environmental context (Eastabrook et al., 2020). However, such responses may be subtle and decoupled from bacterial abundance, leaving the underlying functional adjustments largely unexplored.

Previous work from our group demonstrated that PUA-producing diatoms can alter their chemical profiles depending on the composition of their associated bacterial communities (Hernanz-Torrijos et al., 2023), highlighting a complex bidirectional signaling that necessitates a deeper functional characterization of the bacterial response.

The objective of the present study was to investigate how bacterial assemblages naturally associated with either a PUA-producing (*C. cryptica*) or a non-producing (*P. tricornutum*) diatom host respond to PUA exposure. We specifically aimed to resolve how these communities modulate their metabolic, regulatory, and stress-related functions in response to increasing PUA concentrations—a dimension that remains largely unexplored. To achieve this, we tested three individual PUAs and their mixture across a range of doses, including both ecologically realistic levels and higher concentrations typically used in laboratory assays (Ribalet et al., 2014; Pepi et al., 2017). This dual approach allows us to resolve how bacterial physiological strategies scale with the chemical intensity of their microenvironment.

To bridge the gap between community-level observations and underlying mechanisms, we combined growth-based measurements with comparative proteomic analyses to resolve concentration-dependent and community-specific functional strategies, providing a high-resolution view of bacterial physiological responses to PUA exposure. To our knowledge, this represents the first comparative proteomic analysis of diatom-associated bacterial assemblages exposed to individual and mixed PUAs across ecologically relevant concentration ranges.

## 2 Materials and methods

### 2.1 Chemicals preparation

2*E*,4*E/Z*-heptadienal, 2*E*,4*E/Z*-octadienal and 2*E*,4*E/Z*-decadienal were obtained commercially from Sigma-Aldrich Inc. (Milan, Italy). Working solutions were prepared by diluting the stock in methanol at room temperature. Toxicity of the methanol solvent was tested for bacterial communities and resulted to start above 1 μL of pure methanol per 1 mL of culture (data not shown). Therefore, the amount of aldehyde solutions in each test was kept always below this threshold (0.02% of pure methanol per mL of culture). Furthermore, on previous similar studies by (Pepi et al., 2017) and (Ribalet et al., 2007, 2008), no more than 3% of pure methanol per mL of culture was ever used to ensure that it did not have a toxic effect on the bacterial communities.

### 2.2 Preparation of DOC extracts

To provide a naturally relevant carbon and energy source for the bacterial communities associated with *Cyclotella cryptica* and *Phaeodactylum tricornutum*, we enriched the experimental medium with dissolved organic carbon (DOC) derived directly from exponential-phase *C. cryptica* cultures. This approach was designed to maintain the original metabolic background and community structure of the diatom-associated assemblages, minimizing the artifacts often associated with synthetic media, which can selectively favor specific taxa and bias functional responses independently of PUA exposure.

Large-volume *C. cryptica* cultures were maintained in exponential growth and sequentially processed. For each 2 L culture, algal biomass was removed via Whatman GF/F filtration, followed by 0.22 μm filtration to remove bacteria. That twice filtered volume was eluted through a LiChrolut ® RP C-18 cartridge, previously washed with Milli-Q water, by pumping an Eyela peristaltic pump. This extraction method was chosen to selectively enrich the hydrophobic and moderately polar fractions of the diatom exometabolome, ensuring the recovery of biologically active signaling molecules. The organic carbon was eluted from the cartridge with 4 mL of methanol, collected in glass vials, and evaporated to dryness under reduced pressure. Each organic carbon extract corresponding to 2 L of *C. cryptica* cultures was finally rediluted in 30 mL of autoclaved and 0.22 μm filtered natural seawater. In total, organic matter from 12 L of *C. cryptica* cultures were concentrated in 180 mL of extract. For each experimental culture, 3.5 mL of this solution was added at the beginning of the experiment. This procedure ensured the availability of a stable, chemically complex, and biologically relevant organic carbon pool during the experimental incubations, allowing bacterial responses to PUA exposure to be assessed under non-carbon-limiting and environmentally relevant conditions.

### 2.3 Cultures and experimental design

In this study, two diatom species were used in order to obtain its natural bacterial assemblages: the PUA-producing *C. cryptica* and the non-PUA producing *P. tricornutum* (strain CCAP 1052/1A). *C. cryptica* was freshly isolated from the Atlantic coastal region at the Bay of Cádiz (southwestern Spain). The specie was identified by PCR using the protocol described by (Zimmermann et al., 2011), at the Universitary Institute of Marine Research (INMAR, Cádiz, Spain). *C. cryptica* and *P. tricornutum* stock cultures were maintained by successive inoculations at constant 20 °C, 54 ± 6 μmolquanta m^−2^ s^−1^ in a 14:10 Light: Dark (L: D) cycle in sterile natural seawater enriched with f/2 medium (Guillard, 1975) and silicate. These cultures were filtered through sterilized polycarbonate filters (2 μm, Whatman®-Nucleopore™ Track-Etch Membrane, Maidstone, United Kingdom) to remove algae while preserving the naturally bacterial communities associated to *C. cryptica* (native bacteria, N-community) and *P. tricornutum* (introduced bacteria, I-community) according to (Hernanz-Torrijos et al., 2023) (Supplementary Figure S1).

Bacterial cultures of *C. cryptica* and *P. tricornutum* were grown in a final volume of 35 mL in sterilized Erlenmeyer flasks under artificial light at 54 ± 6 μmolquanta m^−2^ s^−1^ of irradiance and 14:10 (L: D) cycle. They were maintained at 20 °C and orbital agitation at 90 rpm in autoclaved and sterilized 0.22 μm filtered natural seawater enriched with 3.5 mL of concentrated organic matter (Section *2.1 Materials and Methods*). Each experimental flask was inoculated with a bacterial inoculum of 6 × 10^3^ cell mL^−1^ and sealed with cotton wool and parafilm. In order to test whether PUA has a bacteriostatic or bactericidal role, or whether PUA could be used as an alternative source of carbon and energy by bacteria communities, a total of 10 different treatments were applied (*n* = 3 biological replicates per treatment). Two control treatments without aldehydes presence were used: cultures with concentrated organic matter as controls (Ctrl cultures), and cultures with concentrated organic matter and 7 μL of methanol as methanol controls (Ctrl MeOH cultures). For PUA addition treatments, three flasks were inoculated with a final 2*E*,4*E/Z*-heptadienal (HD) concentration of 15 nM, and three flasks were inoculated with a final HD concentration of 500 nM. Independently, same was done with 2*E*,4*E/Z*-octadienal (OD), 2*E*,4*E/Z*-decadienal (DD) and an aldehyde mixture with 2:1:1 molar proportion, that is, 7.5 nM of HD plus 3.5 nM of OD plus 3.5 nM of DD for a 15 nM final MIX concentration; and 250 nM of HD plus 125 nM of OD plus 125 nM of DD for a 500 nM final MIX concentration (Supplementary Figure S1). So, a total of 30 cultures were carried out for each microbial community in order to apply the ten treatments. For cell density quantification, 1 mL of each culture was collected under sterile UV ambient at 2, 6, 24, 48, 72, 96, 144 and 168 h after the PUA or MeOH addition. At the end of the experiment (*t* = 168 h) 15 mL of each culture were used for dissolved organic carbon (DOC) analysis and 3 mL for proteomic analysis. We chose a 168-h exposure timeframe to capture the proteomic signatures of stable acclimatization rather than transient stress responses.

### 2.4 Bacterial growth

Bacterial cell density was quantified by flow cytometry (FC) using a BD ACCURI™ C6 Plus Flow Cytometer. Samples were fixed with a mix of paraformaldehyde (1% final concentration) and glutaraldehyde (0.1% final concentration), frozen in liquid nitrogen and preserved at −80 °C until analysis. For total bacterial counting by IFC, samples were thawed and stained with SYBR Green I nucleic acid gel stain (SYBRGreen) (0.01%) (490–498 nm, S-9430; Sigma Aldrich, Saint Louis, United States; ×10 dilution in DMSO of commercial stock), at dark during 10 min (Gasol and Morán, 2016) before the analysis. This fluorochrome allowed separation of bacterial cells from abiotic particles (e.g., detritus). Also, the absence of diatoms was verified in each culture.

Bacteria growth rates were calculated as *μ* (hours^−1^) according to Equation 1:


$$\mu =\ln\frac{N1}{N0}t$$ (1)

Where *N*_0_ and *N*_1_ represent cell density (cell mL^−1^) at the start and the end of the exponential growth period, and t is the time between measurements (in hours).

In order to detect a possible toxic effect of PUA on bacterial growth, growth inhibition was expressed as relative inhibition and calculated for each treatment by normalizing growth rates (μ, h^−1^) of PUA-exposed cultures (μ₁) to those of the MeOH control cultures (μ₀), according to the following Equation 2:


$$\mu \;(\%\text{of methanol control})=(1-\frac{\mu 1}{\mu 0})\times 100$$ (2)

Where negative values indicate stimulation relative to the MeOH control and positive values indicate inhibition.

### 2.5 Dissolved organic carbon (DOC) analysis

Samples for DOC analysis (15 mL) were collected from the 0.22 μm filtered natural seawater, from the sterilized sea water with post-added concentrate organic carbon from *C. cryptica* cultures, and from each bacterial culture at the end of the experiment (*t* = 168 h). Each sample was filtered through pre-combusted Whatman GF/F filters (450 °C; 4 h), subsequently by 0.22 μm and preserved at 4 °C until the analysis with a total organic carbon and total nitrogen (TOC/NT) Shimadzu analyzer, TOC-L CSN model. The DOC concentration was determined by removal of the inorganic carbon fraction by addition of 2% 2 N HCl and purging of the analyzed sample with air for 10 min.

Carbon consumption rates were estimated by calculating the difference between initial and final DOC concentrations (mg L^−1^), normalized by the duration of the experiment “*t*” (168 h) according to Equation 3: R_DOC_


$$=\frac{\left(\text{initial}\;\mathrm{DOC}-\text{final}\;\mathrm{DOC}\right)}{t}$$ (3)

### 2.6 Proteomic analysis

#### 2.6.1 Protein extraction and digestion

Extraction of proteins was performed by means of ultrasonication with Ultrasonic homogenizer Vibra cell 75,185 (Sonics & Materials, Inc., distributed by Cole-Parmer, United States). A 2 mL mixture of bacterial suspension and lysis buffer (1:1 ratio; Tris 0.2 M, 8% SDS, 80 mM DTT, and Pierce Protease Inhibitor Mini Tablets, EDTA-free, Thermo A32955) was subjected to ultrasonication in three cycles of 20 s each at 75% amplitude, 130 W, and 20 Hz, with samples kept on ice to prevent overheating. Samples were transferred into 15 mL falcon tubes and centrifuged at 15,000 *g* for 5 min. The supernatant (protein extract) was pipetted into fresh tubes, mixed with 4 volumes of cold acetone and stored at −80 °C overnight. Protein pellet was obtained by centrifuging samples at 15,000 *g* for 10 min. Protein pellet was resuspended in 0.2 mL of denaturing buffer (8 M urea, 100 mM Tris–HCl pH 8.5, 5 mM DTT). Protein concentrations were determined using the Qubit Protein Assay kit (Thermo Scientific™, United States) following the manufacturer’s instructions).

Protein digestion was performed using the small-scale FASP protocol described by (Escobar-Niño et al., 2023). Briefly, the protein pellet was reduced for 30 min at room temperature in denaturing buffer. An aliquot of 50 μg protein, diluted in UA buffer (8 M urea, 100 mM Tris–HCl pH 8.5), was added to a 30 kDa cutoff filter (Vivacon 500, VN01H22; Sartorius, Germany) and centrifuged at 14,000 *g* for 10 min. The filter was washed with 450 μL UA buffer at 14,000 *g* for 10 min. Proteins were alkylated for 30 min in the dark with 100 μL CAA solution (55 mM chloroacetamide, 8 M urea, 100 mM Tris–HCl pH 8.5). After centrifugation at 14,000 *g* for 10 min, the filter was washed twice with 450 μL UA buffer. Protein digestion was carried out with 0.5 μg trypsin (Pierce Trypsin MS-Grade, 90,057, Thermo Scientific, United States) in 450 μL dilution solution (100 mM Tris–HCl pH 8.5, 1 mM CaCl₂) at 37 °C overnight. Peptides were collected by centrifuging the filter at 14,000 *g* for 10 min and acidified with 20% TFA (final concentration 0.5%).

#### 2.6.2 LC–MS/MS analysis

Digested Peptide were desalted using C18 solid-phase extraction columns (Pierce 89,873, Thermo Scientific™, Waltham, United States) according to the manufacturer’s instructions. Eluted peptides were dried in a vacuum concentrator (Vacufuge Vacuum Concentrator 5,301 Centrifuge; Eppendorf, Germany) for 40 min. The peptide pellet was resuspended in 25 μL solubilization buffer (0.1% formic acid / 0.02% DDM [n-dodecyl *β*-D-maltoside]), and peptide concentration was measured using a Nanodrop 2000 (Thermo Scientific™, Waltham, United States). Finally, the peptide solution was diluted to 0.5 μg μL^−1^, and 2 μL were used for LC–MS analysis.

Peptides solution were separated using a Vanquish Neo liquid chromatography system (Thermo Scientific, USA) with a PepMap RSLC C18 column (2 μm, 100 Å, 75 μm inner diameter, 50 cm) (Thermo Scientific, United States). Peptides were loaded onto the column and eluted over 43 min using a segmented linear gradient from 1 to 97.5% of solvent B (0 min: 1% B; 0–10 min: 20% B; 10–20 min: 22.5% B; 20–35 min: 45% B; 35–36.1 min: 97.5% B; 36.1–43 min: 97.5% B) (solvent A: 0.1% Formic Acid; solvent B: 80% Acetonitrile, 0.1% Formic Acid) at a flow rate of 300 nL min^−1^.

Mass spectra were acquired by an Orbitrap Exploris 240 mass spectrometer (Thermo Scientific, United States) in data-dependent acquisition (DDA) mode using a TOP15 method. MS spectra were obtained in the Orbitrap Exploris 240 analyzer (Thermo Scientific, United States) in full-scan mode, with a mass range of 350–1,500 m/z, a resolution of 120,000 FWHM, an AGC target of 300%, and a maximum injection time of 60 ms. Precursors were selected with an isolation window of 1.6 m/z. HCD fragmentation was performed with a normalized collision energy of 30%. MS/MS spectra were acquired with a standard AGC target at a resolution of 15,000 FWHM, a maximum injection time of 30 ms, and a fixed first mass of m/z 100. Peptides with a charge state of +1, greater than +6, or with unassigned charge were excluded from MS2 fragmentation. A dynamic exclusion of 30 s prevented repeated selection of precursors.

#### 2.6.3 Proteomic database generation

A custom bacteria protein sequence database was generated using a two-step sequential database reduction strategy. To this aim, in a first search, mass spectra data (Raw data files) were processed using Proteome Discoverer software (version 3.1.1.93). MS/MS spectra were analyzed in sets of ten using the Sequest search engine, independently against databases containing proteins deposited in UniProtKB for the following bacterial groups: Actinomycetes, Alphaproteobacteria, Bdellovibrionales, Candidatus Kaiserbacteriota, Cyanobacteriota, Gammaproteobacteria, Bacteroidia, Pirellulaceae, Cytophagales, Acidimicrobiia, Sphingobacteriales, Verrucomicrobiales, Flavobacteriales, Planctomycetota, Bacillales, Lactobacillales, Balneolales, Fusobacteriales, Saccharimonadales, Chlamydiales, and the Parcubacteria group (databases downloaded on March 24, 2025). The selected bacterial taxa were based on previous OTU analyses conducted by the research group. Proteomes of *Thalassiosira oceanica* and *Thalassiosira pseudonana* were also included to help further exclude proteins belonging to used culture medium. The digestion mode was set as trypsin. The minimum peptide length was set to six amino acids. Methionine oxidation and N-terminal protein acetylation and methionine loss were set as variable modifications. Carbami-domethylation of cysteine residues was set as fixed modification. Peptide scores were recalibrated using the Percolator node, employing a target/decoy approach to estimate the probability of correct identification and control the false discovery rate (FDR). In our consensus workflow, peptides identified with low confidence (FDR < 5%) were included, proteins were required to be identified with at least one peptide, and all peptides were considered regardless of the number of peptide-spectrum matches (PSMs). Shared peptides among proteins were fully included rather than being assigned only to the highest-scoring protein. The FASTA files generated for in each search group were unified into a single database using the “Compile Fasta Database” tool in Proteome Discoverer. This taxon-specific FASTA generated in this step was then used for a second search in Proteome Discoverer (version 3.1.1.93), using the same criteria but increasing peptide identification confidence to medium (FDR > 5%).

The FASTA files generated for bacteria and diatoms proteins during the second step of database reduction were used in the final search alongside the contaminant database.

#### 2.6.4 Protein identification

Protein identification and quantification were performed using Proteome Discoverer 3.1.1.93) (Orsburn, 2021). Protein identification was carried out using the Processing Workflow PWF_Hybrid_Precursor_Quant_and_LFQ_MPS_SEquestHP_Percolator and the Consensus Workflow CWF_Comprehensive_Enhanced_Annotation_LFQ_and_Precursor_Quan. Raw data were first calibrated with the Spectrum File RC node, followed by feature detection using the Minora Feature Detector node. Digestion was set to trypsin with a minimum peptide length of six amino acids. The following modifications were considered: methionine oxidation, N-terminal protein acetylation, and methionine loss as variable modifications, and carbamidomethylation of cysteine as a fixed modification. Peptides were retained if they passed a false discovery rate (FDR) ≤ 1%. Quantitation was performed using the Precursor Ion Quantifier node, considering peptides classified as unique + razor, with precursor abundances based on intensity. Only features detected in 100% of replicates were used. Protein abundances were calculated by summing the abundances of peptides, and values were normalized by total peptide abundance per sample. Hypothesis testing was performed using *t*-test background based. Adjusted *p*-values for differential protein abundance were calculated by Proteome Discoverer using the Benjamini-Hochberg procedure (Benjamini and Hochberg, 1995) to control the false discovery rate at each tested ratio. Databases used for protein identification included the ones generated in the previous step, supplemented with a list of common contaminant proteins from The Global Proteome Machine (TheGPM).

Exclusive proteins were defined as those detected in all three biological replicates of one condition and not identified in any of the three replicates of the compared condition, whereas absent proteins were defined as those not identified in any replicate of one condition but detected in all replicates of the compared condition. For differential abundance analysis, proteins were considered upregulated in condition A when the Abundance Ratio (A/B) was ≥2 and downregulated when it was ≤0.5; conversely, proteins were considered upregulated in condition B when the ratio was ≤0.5 and downregulated when it was ≥2. In all cases, statistical significance was defined as an adjusted *p*-value ≤0.05.

Finally, proteins were functionally annotated based using Omicsbox v3.0.29 (Buchfink et al., 2021) for the Basic Local Alignment Search Tool (BLAST), Gene Ontology (GO) mapping and annotation; UniProt; and subsequent KEGG and BRITE classification (Gotz et al., 2008).

#### 2.6.5 Relative taxonomy analysis

Prophane^1^ (Schiebenhoefer et al., 2020) was used in expert mode to allow modification of default parameters, Protein group files generated from the database search in Proteome Discoverer were imported into Prophane, and samples were organized according to their corresponding experimental groups. The protein FASTA required as input for Prophane was generated in Proteome Discoverer from the final protein identification step. Quantification was based on normalized protein abundances obtained in Proteome Discoverer. Taxonomic annotation was performed using DIAMOND (BlastP mode) against the UniProtKB database (Swiss-Prot and TrEMBL sections). Taxonomic assignments were inferred using a lowest common ancestor (LCA) approach implemented in Prophane, applying the “LCA per group” setting, ignoring unclassified proteins and those with less than 3 number of annotation.

Prophane results were filtered using a Python-based algorithm employing pandas, argparse, os, tqdm, logging, and sys libraries. This algorithm processes the output files generated by Prophane, performing individual recalculation for each group, retaining only normalized abundance values (quant:sample name (mean)) from protein groups with quantification data in at least 3/3 (proteins quantified in all replicates of each condition) of the analysed replicates. Values for those not meeting this criterion were set to 0. From the recalculated data, input files for Krona plot construction were generated. Visualization of the results was carried out using KronaTools (Ondov et al., 2011), a set of scripts designed to generate interactive hierarchical data plots. These files were processed to produce interactive Krona charts, allowing visualization of the taxonomic distribution for each analysed group.

### 2.7 Statistical analysis

Cell densities and bacterial growth rates did not conform to a normal distribution (Shapiro–Wilk test, *p* < 0.05). Accordingly, non-parametric tests were applied. Differences among treatments were assessed using Kruskal–Wallis tests, followed by Dunn’s *post hoc* pairwise comparisons when appropriate. Pairwise comparisons between treatments and the methanol control (Ctrl MeOH) were performed using Wilcoxon rank-sum tests. *p*-values were adjusted for multiple testing using the Holm (Holm–Bonferroni) method.

Statistical analyses of relative inhibition percentages were performed separately for each PUA concentration. Depending on data distribution, differences among treatments were evaluated using either Kruskal–Wallis or one-way ANOVA tests, followed by Dunn’s or Tukey’s post hoc tests, respectively. Deviations from the Ctrl MeOH were assessed using one-sample tests (one-sample Wilcoxon signed-rank test or one-sample *t*-test, depending on data normality). All statistical analyses were performed using R version 4.5.2.

## 3 Results

### 3.1 Bacterial growth

**Figure 1:**
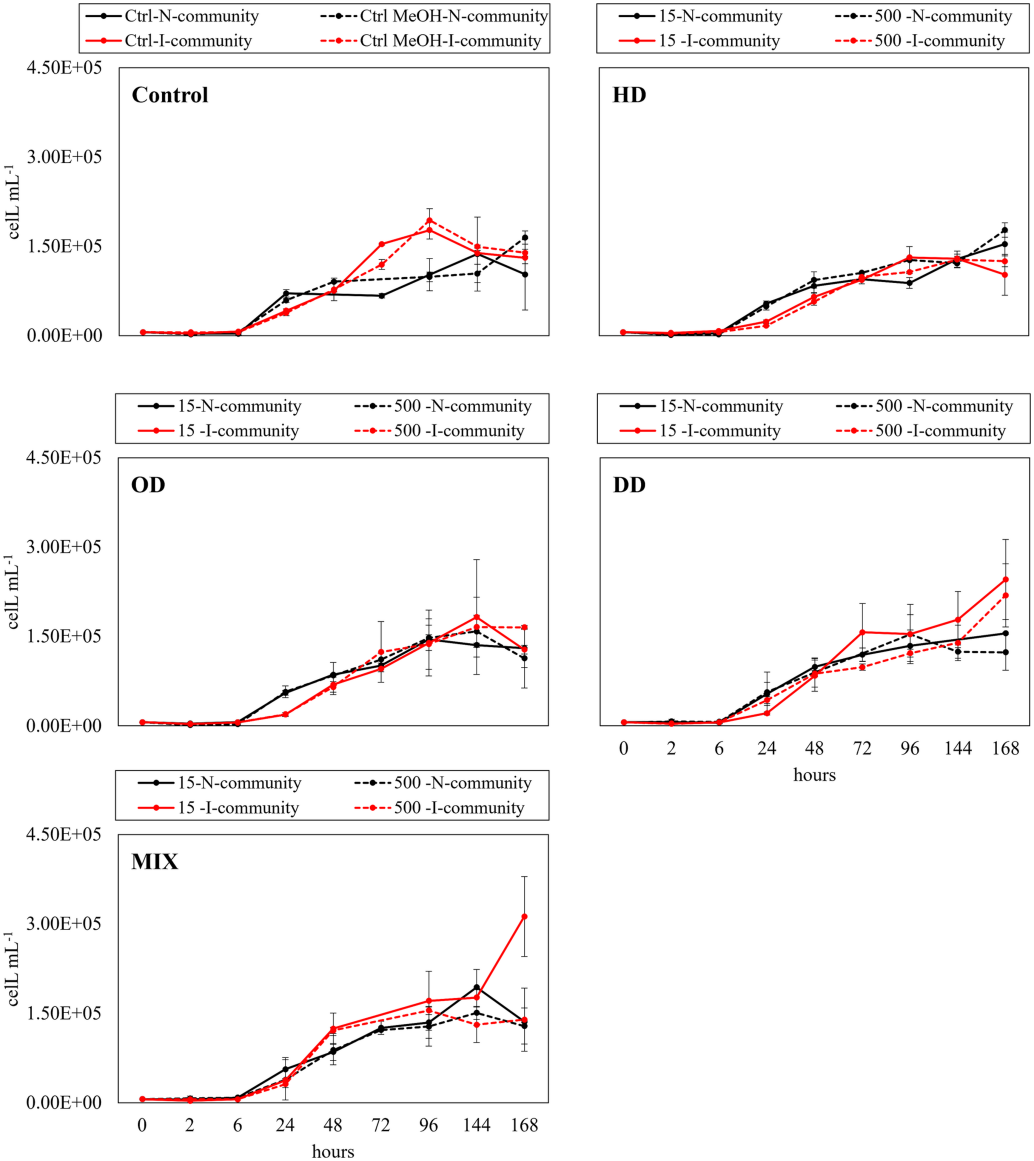
Growth dynamics of native and introduced bacterial communities under PUA exposure. Temporal evolution of cell density (cells mL-1) for native (N-community, black lines) and introduced (I-community, red lines) bacterial assemblages across the experimental treatments. Data are presented as means ± standard deviation (*n* = 3 biological replicates). Treatment key: Ctrl, control cultures; Ctrl MeOH, methanol control; HD, 2*E*,4*E/Z*-heptadienal; OD, 2*E*,4*E/Z*-octadienal; DD, 2*E*,4*E/Z*-decadienal; MIX, mixture of HD, OD, and DD in a 2:1:1 molar proportion.

**Figure 2:**
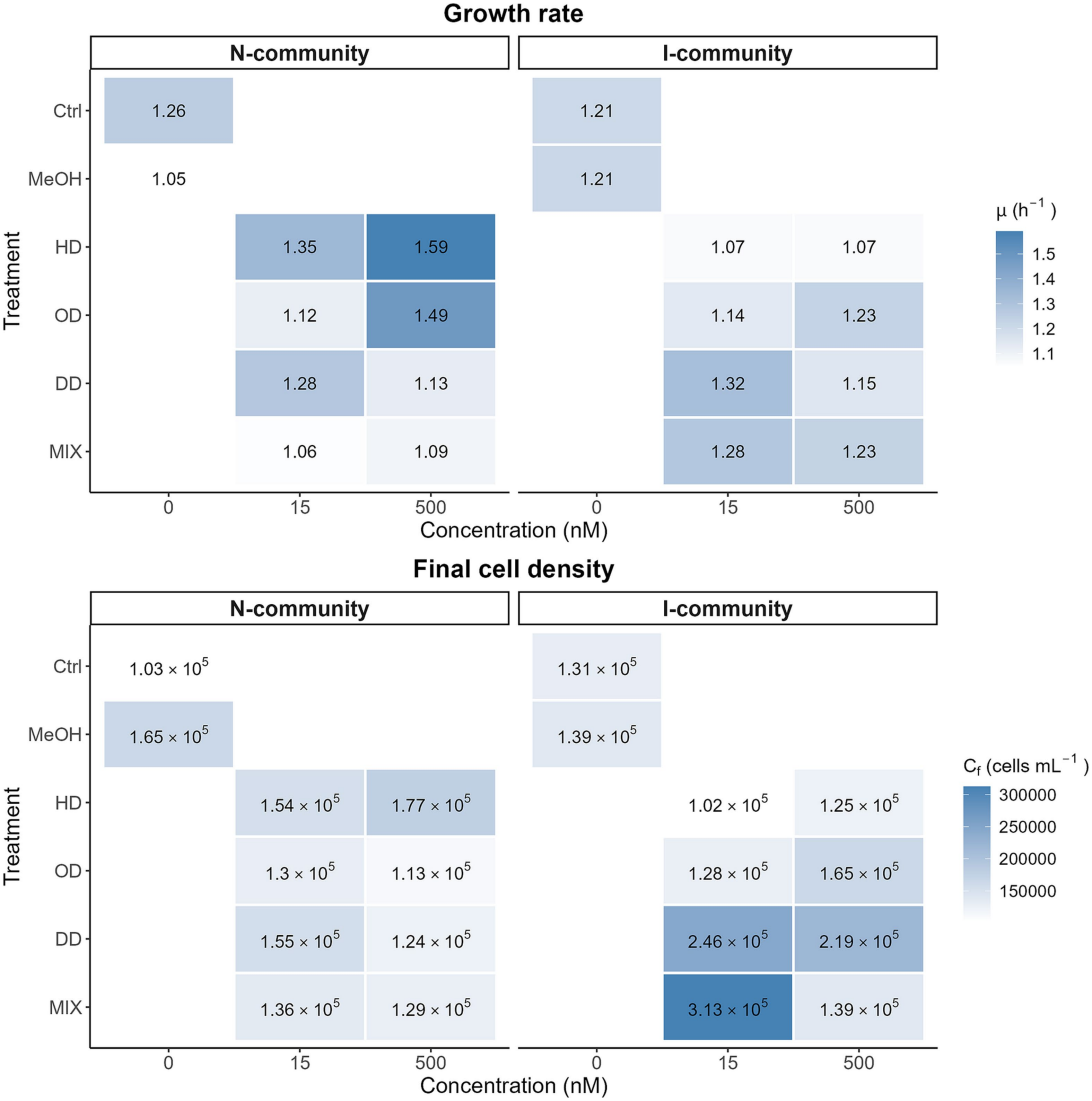
Heatmap representation of bacterial growth rate and final cell concentration across treatments and communities. Heatmaps show the mean growth rate (μ) and final cell density (*C_f_*) for each treatment and concentration in the native (N-community) and introduced (I-community) bacterial assemblages across all the treatments. Values are averaged across biological replicates (*n* = 3 biological replicates). Treatment key: Ctrl, control cultures; Ctrl MeOH, methanol control; HD, 2*E*,4*E/Z*-heptadienal; OD, 2*E*,4*E/Z*-octadienal; DD, 2*E*,4*E/Z*-decadienal; MIX, mixture of HD, OD, and DD in a 2:1:1 molar proportion.

Initial cell densities were established at 6,000 cell mL^−1^ across all cultures. At the end of the experiment, cell densities increased to a maximum of 1.77E+05 cell mL^−1^ and 3.13E+05 cell mL^−1^ in N-community and I-community, respectively. Overall, all cultures grew exponentially and reached similar final densities (Figures 1, 2), except in the I-cultures under 15 nM of DD and MIX treatments, which showed the highest cell densities among all cultures (Figures 1, 2; Supplementary Table S1). Despite this trend, differences in final cell densities were not statistically significant (Kruskal–Wallis test, *p* > 0.05). Similarly, no significant differences in growth rates were detected between treatments and control cultures (Ctrl MeOH) (Wilcoxon test, *p* > 0.05) (Supplementary Table S1).

**Figure 3:**
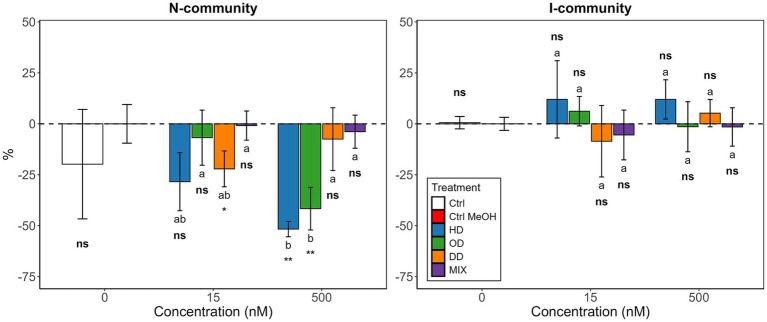
Relative growth inhibition of native and introduced bacterial communities under PUA exposure. Relative inhibition percentages are shown for native (N-community) and introduced (I-community) assemblages across all experimental treatments. Data represent means ± standard deviation (*n* = 3 biological replicates). Positive values indicate growth inhibition relative to the Ctrl MeOH, whereas negative values signify growth stimulation; values near zero reflect no detectable effect. Different letters denote significant differences among PUA treatments (one-way ANOVA followed by Tukey’s *post hoc* test, *p* ≤ 0.05). Asterisks indicate significant differences relative to the methanol control (Ctrl MeOH) based on two-sample *t*-test: ns, not significant; * *p* < 0.05; ** *p* < 0.01; *** *p* < 0.001. Treatment key: Ctrl, control cultures; Ctrl MeOH, methanol control; HD, 2*E*,4*E*/*Z*-heptadienal; OD, 2*E*,4*E*/*Z*-octadienal; DD, 2*E*,4*E*/*Z*-decadienal; MIX, mixture of HD, OD, and DD in a 2:1:1 molar proportion.

In the N-community, relative inhibition percentages remained negligible or negative across all treatments. Notably, even at the maximum PUA concentration (500 nM), no growth inhibition was observed; instead, a slight stimulatory effect was recorded (Figure 3). This was consistent with the similarity of growth curves (Figure 1) and comparable final cell densities across treatments (Figure 2).

Nevertheless, analysis of inhibition percentages revealed a concentration- and treatment-dependent response in N-community. Treatments were separated into three statistically distinct groups (one-way ANOVA followed by Tukey’s *post hoc* test, *p* ≤ 0.05; Figure 3), with OD and MIX at 15 nM and DD and MIX at 500 nM showing the lowest percentages (group “a”), HD and DD at 15 nM displaying intermediate responses (“ab”), and HD and OD at 500 nM exhibiting the highest (“b”). In addition, at 500 nM, inhibition values for HD (*p*-value <0.01) and OD (*p*-value <0.01) differed significantly from the Ctrl MeOH, whereas DD and MIX did not (two-sample *t*-test, *p*-value >0.05) (Figure 3).

I-community did not show significant differences among treatments at any of the concentrations tested (one-way ANOVA, Tukey *post hoc*, *p*-value >0.05). No differences were found when comparing with Ctrl MeOH (two-sample *t*-tests, *p*-value >0.05). Although relative inhibition values suggested a slight inhibitory trend for HD and OD, particularly at 15 nM, these effects were not statistically supported (Figure 3).

Overall, relative inhibition analyses revealed a differential response to PUA between native and introduced bacterial communities.

### 3.2 Dissolved organic carbon and bacterial carbon consumption rates

Final dissolved organic carbon (DOC) concentrations in all PUA-treated and Ctrl MeOH cultures remained above 50 mg L^−1^, whereas in control cultures (Ctrl), DOC values dropped below 20 mg L^−1^ (Supplementary Figure S2). At the end of the experiment, DOC remained available in all treatments.

Positive DOC consumption rates were observed in all treatments, indicating net organic carbon consumption by both bacterial communities throughout the experiment. Both bacterial communities actively utilized at least part of the organic carbon present in the medium under all experimental treatments. Across all treatments, N-community consistently showed higher carbon consumption rates than I-community. In N-community, all PUA treatments were associated with significantly higher DOC consumption rates compared to control conditions (Ctrl and Ctrl MeOH) (one-way ANOVA, Tukey’s HSD post hoc test, *p* < 0.05). By comparison, in I-community, DOC consumption rates under Ctrl MeOH and PUA treatments were higher than those observed in Ctrl cultures (one-way ANOVA, Tukey’s HSD post hoc test, *p* < 0.05) (Supplementary Figure S3).

### 3.3 Proteomic analysis

To explore treatment-specific proteomic response across both bacterial communities (N- and I-community), proteomic analysis was performed on a selected subset of treatments embracing low vs. high PUA concentration (15 vs. 500) and individual vs. mixed PUA treatment (DD vs. MIX). These treatments were also selected based on consistent trends observed in the growth curves particularly in the I-community, where DD (at both 15 and 500 nM) and MIX (at 15 nM) reached higher final cell densities compared to other treatments and differed from the patterns observed in the N-community. The mass spectrometry proteomics data have been deposited in the ProteomeXchange Consortium via the PRIDE partner repository (Deutsch et al., 2023; Perez-Riverol et al., 2025), with the dataset identifier PXD075100.

**Figure 4:**
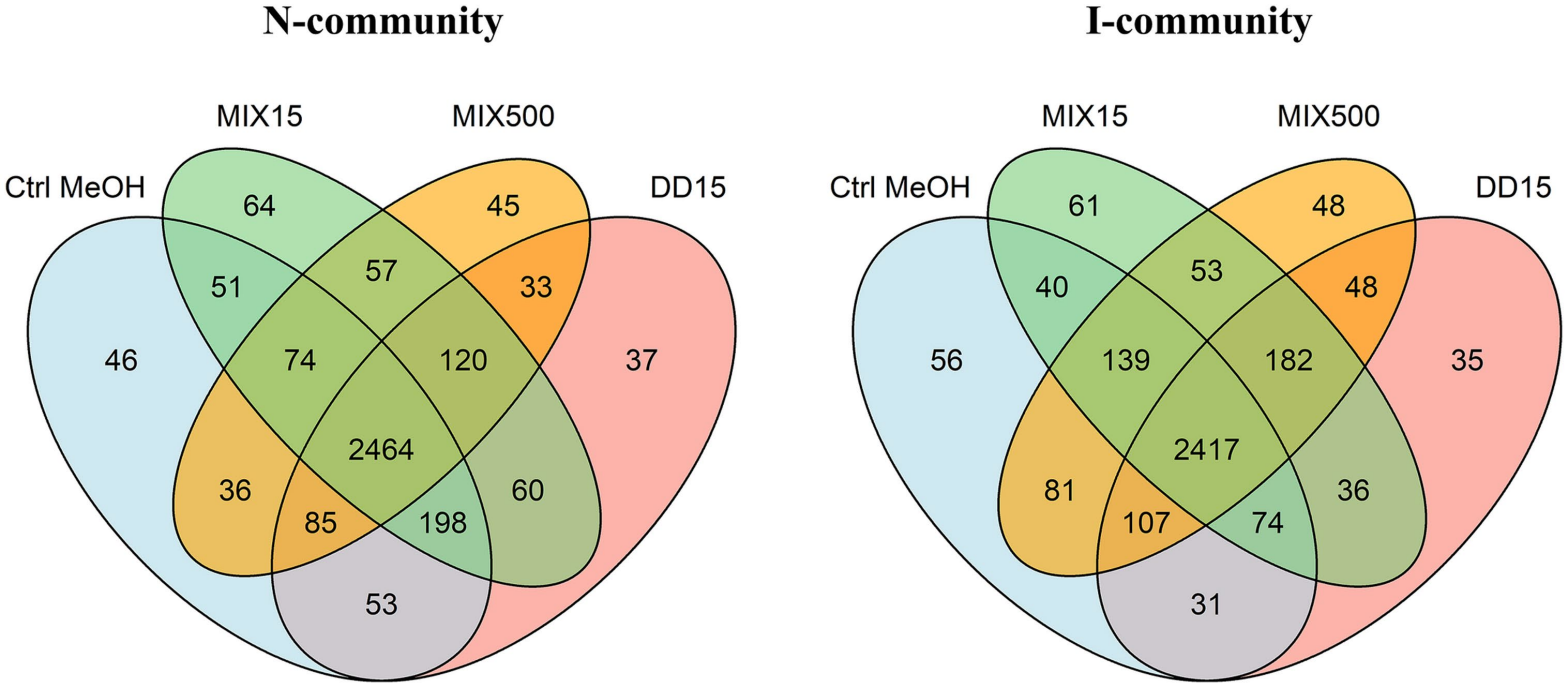
Comparative analysis of the core and treatment-specific proteomes in native and introduced bacterial communities. Venn diagrams illustrate the distribution of proteins across the four experimental treatments (Ctrl MeOH, DD15, MIX15, and MIX500) for both native (N-community) and introduced (I-community) assemblages. Overlapping regions represent the core proteome (shared across all treatments) and proteins common to specific PUA exposures, while non-overlapping areas identify treatment-specific proteins. Data are based on the presence or absence of protein detection (*n* = 3 biological replicates).

Across N- and I-communities, the global proteomic survey identified a total of 3,781 proteins without applying any filtering criteria (Supplementary Table S2A). Of these, 3,736 remained after removing diatom-derived proteins from the culture medium (Supplementary Table S2B). Among them, 2,464 proteins were shared between the four experimental treatments (Ctrl MeOH, DD15, MIX15 and MIX500) in N-community, whereas 2,417 proteins were consistently detected across the four treatments in I-community (Figure 4). These proteins define the core proteome shared among treatments within each bacterial community.

To evaluate treatment-specific proteomic responses in the bacterial communities, detected proteins were classified into four categories: absent, exclusive, upregulated and downregulated, following the criteria described in Section *2.6.4 Materials and Methods*. This classification enabled both quantitative and qualitative comparison of proteomic changes among experimental treatments and between bacterial communities. This approach allowed us to independently assess the responses of native (N-community) and introduced (I-community) bacterial assemblages to increasing PUA concentrations, and subsequently to disentangle community-specific proteomic patterns.

Across all pairwise comparisons, including treatment-to-treatment comparisons within each bacterial community, and community-to-community comparisons within each treatment, each category comprised more than one hundred proteins (Supplementary Tables S3A,B). This high number of differentially classified proteins indicates that PUA exposure and concentration elicited a substantial restructuring of the bacterial proteome across multiple comparative levels, rather than marginal or protein-specific effects. These broad comparative patterns establish the basis for the subsequent filtering steps and for the detailed, community-specific functional analyses presented below.

#### 3.3.1 N-community proteome response to concentration

**Figure 5:**
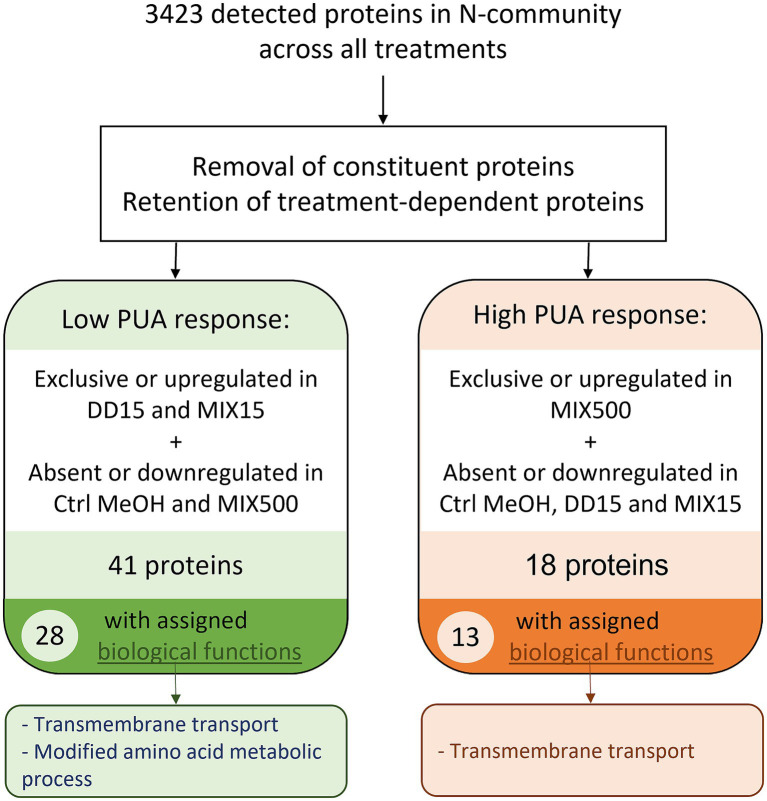
Conceptual framework of the proteomic filtering strategy for identifying concentration-dependent responses. Schematic representation of the bioinformatic pipeline used to isolate specific protein subsets in N-community. The filtering process prioritizes proteins showing differential expression patterns across increasing PUA concentrations, distinguishing between baseline metabolic functions and specialized stress-response mechanisms. Only Gene Ontology (GO) biological process categories accounting for >15% of the functionally annotated proteins are shown.

Within the total set of 3,423 proteins detected in N-community, 41 proteins were specifically associated with DD15 and MIX15 treatments, being exclusive or upregulated under these conditions and absent or downregulated in Ctrl MeOH and MIX500 treatments (Supplementary Table S4A). In this and all subsequent comparisons, Ctrl MeOH was systematically included as a reference condition to exclude constitutively expressed proteins of the community and to ensure that the resulting protein subsets reflected responses specifically associated with PUA exposure, rather than baseline proteomic features. These proteins therefore define a subset selectively expressed in response to low PUA exposure. Of the 41 proteins identified, 28 received annotations after the functional analyses based on UniProt and subsequent KEGG classification and GO mapping (Figure 5; Supplementary Figure S4; Supplementary Table S4B).

Functionally, the low PUA–associated subset was predominantly linked to transport and membrane-associated processes and to oxidoreductase activity, including proteins involved in glutathione-related metabolism and xenobiotic biodegradation. Regulatory components were also represented, encompassing signal transduction, transcriptional control and central carbon metabolism. In addition, proteins associated with protein folding, ribosome-related functions, chromosome organization and sporulation were identified within this subset.

A second subset of treatment-specific proteins was identified in N-community under MIX500. In total, 18 proteins were exclusive or upregulated under MIX500 compared to Ctrl MeOH, DD15 and MIX15 (Supplementary Table S5A), thus defining a subset selectively expressed in response to high PUA exposure. Of these, 13 proteins were assigned biological functions (Figure 5; Supplementary Table S5B). Functionally, this high PUA–associated subset was mainly characterized by proteins linked to cellular communication and regulatory signaling, including quorum sensing–related components, together with proteins involved in signal transduction and transcriptional regulation. Antimicrobial resistance–associated processes and ABC-type transport systems were also represented. In addition, proteins related to cell–cell adhesion and biofilm formation were identified, as well as components associated with cellular processes such as flagellar assembly and sporulation.

Overall, the treatment-specific protein subsets identified in N-community under low (DD15/MIX15) and high (MIX500) PUA exposure exhibited clearly distinct functional profiles. Low PUA exposure was mainly associated with oxidoreductase and glutathione/xenobiotic-related metabolic processes, together with transport and regulatory functions. In contrast, the MIX500 subset was characterized by quorum sensing– and cyclic di-GMP–associated signaling, antimicrobial resistance–related processes and ABC-type transport systems. These contrasting patterns indicate a concentration-dependent shift from predominantly metabolic and redox-associated responses at low PUA levels to regulatory and community-related processes under high PUA exposure, reflecting a concentration-dependent reorganization of the N-community proteome.

#### 3.3.2 I-community proteome response to concentration

**Figure 6:**
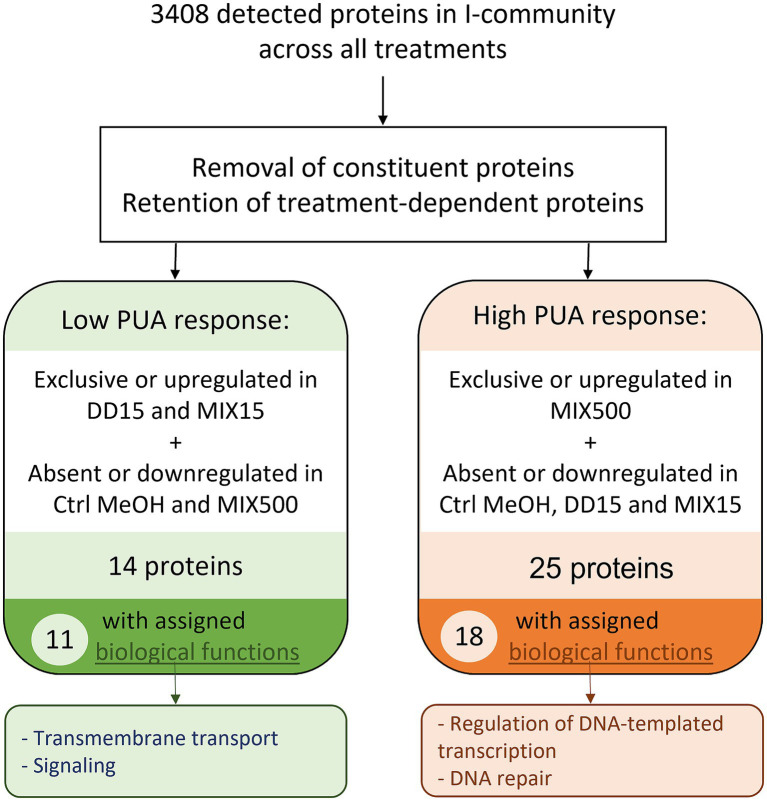
Conceptual framework of the proteomic filtering strategy for identifying concentration-dependent responses. Schematic representation of the bioinformatic pipeline used to isolate specific protein subsets in I-community. The filtering process prioritizes proteins showing differential expression patterns across increasing PUA concentrations, distinguishing between baseline metabolic functions and specialized stress-response mechanisms. Only Gene Ontology (GO) biological process categories accounting for >15% of the functionally annotated proteins are shown.

Within the total set of 3,408 proteins detected in I-community, 14 proteins were specifically associated with DD15 and MIX15 treatments, being exclusive or upregulated under these conditions and absent or downregulated in Ctrl MeOH and MIX500 (Supplementary Table S6A). These proteins define a subset selectively expressed under low PUA exposure in I-community. Of these, 11 were assigned biological functions (Figure 6; Supplementary Figure S4; Supplementary Table S6B).

Functionally, the low PUA–associated subset in I-community was predominantly related to metabolic processes, particularly carbohydrate metabolism and the biosynthesis of nucleotide sugars and secondary metabolites. Proteins linked to environmental information processing were also represented, including membrane transport and signal transduction functions. In addition, regulatory components and proteins associated with cellular surface-related processes, such as biofilm-associated functions, were identified, together with elements involved in genetic information processing, including translation- and RNA-related functions.

A second subset of treatment-specific proteins was identified in I-community under MIX500. In total, 25 proteins were exclusive or upregulated under MIX500, while being absent or downregulated in Ctrl MeOH, DD15 and MIX15 treatments (Supplementary Table S7A). These proteins therefore define a subset selectively expressed in response to high PUA exposure. Of these proteins, 18 received annotations after the functional analyses (Figure 6; Supplementary Table S7B). Functionally, this high PUA–associated subset was mainly linked to metabolic processes, including pathways related to carbohydrate metabolism, fatty acid *β*-oxidation, methylation and glyoxylate/dicarboxylate metabolism. Proteins involved in genome maintenance, DNA replication and repair, and transcriptional control were also represented within this subset. Finally, several components were related to cellular processes, including motility, cell–cell adhesion, antibiotic biosynthetic functions and membrane-associated transport systems. Proteins involved in protein modification and metabolic regulation were likewise detected within this subset.

The two treatment-specific protein subsets identified in I-community under low (DD15/MIX15) and high (MIX500) PUA exposure differed in their functional composition. Low PUA exposure was primarily associated with environmental information processing and regulatory functions, particularly signal transduction and membrane transport, together with carbohydrate-related metabolic processes. In contrast, high PUA exposure was characterized by a stronger representation of genetic information processing, including DNA replication and repair and transcription-associated functions, alongside diverse metabolic pathways such as carbohydrate metabolism, fatty acid β-oxidation and methylation. These patterns indicate a concentration-dependent restructuring of functional responses in I-community, with distinct protein subsets associated with increasing PUA levels and treatment composition.

#### 3.3.3 Comparative proteomic responses between bacterial assemblages

Treatment-specific proteomic responses differed between N- and I-communities across PUA exposure conditions. Although both assemblages exhibited distinct protein subsets under low and high PUA exposure, the functional composition of these subsets differed markedly between communities.

Under low PUA exposure (DD15 and MIX15), contrasting functional profiles were observed. In N-community, the response was primarily associated with metabolic and redox-related processes, including oxidoreductase activity and glutathione/xenobiotic metabolism, together with transport and regulatory functions (Supplementary Table S4B). In contrast, I-community showed a response mainly characterized by environmental information processing and regulatory functions, including membrane transport, signal transduction and transcriptional regulation, accompanied by carbohydrate-related pathways (Supplementary Table S6B). Direct comparison of the low PUA–associated subsets revealed minimal overlap between communities. Only three shared proteins were detected: a sodium:dicarboxylate symporter, an uncharacterized sulfatase and an uncharacterized membrane protein (Supplementary Tables S4B and S6B). This limited overlap suggests that low PUA exposure may trigger a small core response independent of community composition, while most functional adjustments remain community-specific.

Differences between bacterial assemblages became more pronounced under high PUA exposure (MIX500). In N-community, the high PUA subset was mainly characterized by signaling and communication-related functions, including quorum sensing and cyclic di-GMP–associated processes, together with antimicrobial resistance–linked components, together with transport systems and metabolism linked to cofactors and secondary metabolites (Supplementary Table S5B). Conversely, I-community exhibited a response dominated by genetic information processing, particularly DNA repair and transcription-associated functions, alongside by energy-related metabolic pathways (Supplementary Table S7B). Importantly, no shared proteins were detected between N- and I-community under high PUA exposure (MIX500), indicating that proteomic responses at elevated PUA concentrations were entirely community-specific (Supplementary Tables S5B and S7B).

Regarding relative taxonomy analysis, The N-community maintained stable proteomic and taxonomic profiles across all tested concentrations, with highly conserved taxonomic structures (Supplementary Figure S5). In the I-community, 15 nM PUA induced moderate taxonomic shifts, characterized by an increased proportion of Gammaproteobacteria and a reduction in the “various” protein fraction. At 500 nM, the I-community proteomic and taxonomic profiles were similar to those of the control group, indicating the absence of the intermediate shifts observed at lower doses.

## 4 Discussion

Our results demonstrate that exposure to 15 and 500 nM PUA, whether administered individually or as a mixture, does not result in strong inhibition of overall community growth rates, although relative inhibition values indicate a stimulatory response in the N-community and a moderate inhibitory effect in the I-community (Figures 1, 3; Supplementary Table S1). Across all treatments, cell densities and growth rates did not differ significantly from Ctrl MeOH cultures (*p* > 0.05; Supplementary Table S1, Figure 1). These findings challenge the traditional view of PUAs as purely bactericidal or bacteriostatic compounds (Bisignano et al., 2001; Adolph et al., 2004; Pepi et al., 2017), aligning with emerging evidence that their biological impact is highly context-dependent rather than universally inhibitory (Balestra et al., 2011; Edwards et al., 2015; Wu et al., 2021). A critical finding was that carbon availability did not constrain bacterial activity; thus, the proteomic shifts observed are not artifacts of nutrient limitation. N-communities showed significantly higher DOC consumption under PUA exposure (*p* < 0.05), suggesting a metabolic priming effect. In contrast, I-communities showed no such stimulation (Supplementary Figure S3). This divergence points to a community-history-dependent metabolic adjustment. While we cannot definitively determine if PUAs served as a direct carbon source, their contribution is likely minor compared to the background DOC—a scenario that accurately reflects the chemically complex conditions of the natural phycosphere.

Against this background of stable growth and non-limiting carbon availability, proteomic analyses revealed distinct yet constrained adjustments in both communities across PUA concentrations.

In N-community, PUA exposure triggered a coordinated, stability-oriented proteomic response rather than a damage-control program. At low concentrations, the proteome was dominated by redox-balancing and detoxification machinery (e.g., glutathione metabolism and xenobiotic processing (Supplementary Table S4B), allowing bacteria to maintain homeostasis without growth trade-offs (Sporer et al., 2017; Addorisio et al., 2022). As concentrations increased, the functional emphasis shifted toward high-level regulatory systems, including quorum sensing and cyclic di-GMP signaling (Supplementary Table S5). These regulatory systems are widely recognized as central integrators of environmental cues, coordinating community-level behaviors and adaptive responses to fluctuating chemical conditions (Hmelo, 2017; Jenal et al., 2017; Mukherjee and Bassler, 2019; Fei et al., 2020). Notably, no activation of DNA repair, protein folding, or other classical damage-control mechanisms were detected across the concentration range. The absence of canonical stress signatures suggests that PUAs are perceived by adapted bacteria as predictable environmental cues rather than acute stressors. This ‘pre-programmed’ response likely stems from co-evolutionary processes within the phycosphere of PUA-producing diatoms (Di Costanzo et al., 2023).

Ecologically, this response pattern is consistent with long-term exposure to PUA, as these bacterial assemblages are naturally associated with a PUA-producing diatom. In their natural niche, PUA likely represent predictable components of the phycosphere rather than acute stressors. Recurrent exposure within this chemically structured microenvironment may favor the selection of regulatory and redox-balancing traits that enable stable physiological accommodation without triggering extensive stress responses. This interpretation aligns with previous studies reporting intrinsic tolerance to PUA among diatom-associated bacteria, potentially arising from physiological adaptation or co-evolutionary processes with their phytoplankton hosts (Ribalet et al., 2008; Amin et al., 2012; Edwards et al., 2025). In this context, the constrained and stability-oriented proteomic profile observed in N-community may reflect accommodation to recurrent host-derived metabolites rather than acute stress responses. Within this context, PUA function less as toxic compounds or nutritional substrates and more as chemical cues contributing to the structuring of the phycosphere (Shibl et al., 2020).

In contrast, I-community exhibited a stark, dose-dependent functional reorganization. At low concentrations, the proteomic profile was dominated by proteins involved in environmental information processing and regulatory functions, including membrane transport systems, two-component signaling pathways, transcriptional regulators, carbohydrate metabolism, and cell surface-associated processes (Supplementary Table S6A). This pattern suggests that PUA were perceived as manageable environmental signals, enabling physiological adjustment without triggering extensive maintenance or stress-related programs and thus avoiding unnecessary energetic costs (Mori et al., 2016; O’Brien et al., 2016). Such regulatory flexibility may have supported metabolic efficiency and resource utilization, thereby sustaining continued growth, consistent with the higher final cell densities observed under DD15 and MIX15 treatments (Figures 1, 2; Supplementary Table S1). By contrast, exposure to high PUA concentrations (MIX500) induced a pronounced qualitative shift in the proteomic profile of I-community. Proteins related to genetic information processing became predominant, particularly those related to DNA replication and repair, transcription-associated functions and ribosome-related processes (Supplementary Table S7B). Such pathways are typically induced under acute oxidative or chemical stress as part of canonical bacterial stress responses (Imlay, 2013). This response was accompanied by the activation of energetically demanding metabolic pathways, indicating a substantial reallocation of cellular resources toward maintenance functions (Scott et al., 2010; Hui et al., 2015). Together, these changes reflect a transition from a signal-driven regulatory response at moderate exposure to a maintenance-oriented survival strategy under elevated chemical stress, reflecting a late but substantial physiological reorganization (Barak-Gavish et al., 2023).

Accordingly, this shift toward cellular maintenance at the expense of biosynthetic and growth-associated functions likely underlies the reduced cell densities observed at 500 nM compared to 15 nM under MIX treatment (Figures 1, 2; Supplementary Table S1).

The contrasting proteomic responses observed between the two bacterial communities reveal the deployment of distinct functional strategies in response to PUA exposure. Bacteria are not passive recipients of phytoplankton exudates but actively adjust their physiology in response to chemical cues (Amin et al., 2015; Landa et al., 2017; Seymour et al., 2017; Mühlenbruch et al., 2018). They respond and acclimate dynamically modulating metabolic and regulatory pathways according to environmental context. In this comparative framework, N-community—naturally associated with a PUA-producing diatom—appear to rapidly recognize the presence of these aldehydes and deploy a consistent and concentration-independent proteomic response. This early and stable activation of specific functional pathways may reflect a “pre-programmed” response shaped by long-term exposure to host-derived PUA, allowing coexistence without promoting enhanced growth. Such stability-oriented strategies are consistent with the maintenance of symbiotic equilibrium through specialized regulatory systems and redox-balancing traits, ensuring survival within the phycosphere (Zhu et al., 2021). Limited proteomic and taxonomic changes across concentrations in the N-community indicate a stable, regulation-oriented response consistent with efficient physiological adjustment. Taxonomic profiles remained highly conserved, supporting a community-wide maintenance of functional structure (Supplementary Figure S5). In contrast, the I-community showed moderate taxonomic shifts at 15 nM, particularly an increased contribution of Gammaproteobacteria accompanied by a decrease in the “various” fraction (Supplementary Figure S5). Since this category includes highly conserved proteins with low taxonomic resolution, these changes likely reflect a redistribution of taxon-specific protein signals rather than a constitutive response of the whole community. Accordingly, these patterns cannot be unambiguously interpreted as per-taxon activation or repression, but rather as a change in the types of proteins detected within each taxonomic group, which may underlie a more specific response to moderate doses of PUA. At 500 nM, the I-community showed a proteomic and taxonomic profile similar to controls, suggesting a loss of this intermediate response and the onset of broader functional disruption under higher chemical pressure.

PUAs may influence bacterial colonization dynamics, by shaping the chemical landscape surrounding the diatom cell, promoting adapted taxa while constraining less tolerant populations, thereby contributing to selective community assembly in the phycosphere. Over ecological timescales, such chemically mediated selection pressures may favor the retention of genetic and proteomic traits that enable compatibility within PUA-rich microenvironments. In this context, the stability-oriented response observed in N-community and the stress-dependent reorganization detected in I-community illustrate how prior ecological history conditions bacterial physiological responses to diatom-derived PUAs.

The lack of shared activated proteins between N- and I-communities at high PUA concentrations (Supplementary Tables S5B and S6B) highlights the community-specific nature of the response. Our data suggest that PUAs act as a selective filter in the phycosphere: promoting stability-based strategies in adapted taxa (N) while imposing significant metabolic burdens on unexposed populations (I). This chemically mediated selection pressure likely shapes community assembly over ecological timescales. Crucially, this level of mechanistic resolution was only achievable through comparative proteomics, revealing a hidden layer of functional reorganization conditioned by ecological history and taxonomic composition that remains invisible to traditional abundance-based measurements.

Although our experimental design does not directly assess community assembly, the divergent proteomic strategies observed in our study provide a mechanistic explanation for the microbial successions documented during phytoplankton blooms in the ocean. Our results suggest that PUAs act as a selective filter: while some bacterial clades may be inhibited, others—like the communities analyzed here—undergo a functional reprogramming that secures their ecological fitness within the phycosphere. This “proteomic plasticity” ensures that key biogeochemical processes, such as organic matter remineralization, continue even under high concentrations of diatom-derived oxylipins. Consequently, the ability of specific bacterial assemblages to switch from acute stress response to stable metabolic acclimatization at 168 h highlights the role of chemical signaling as a driver of community structure, ultimately influencing the efficiency of the biological carbon pump during the decline of diatom blooms.

## 5 Conclusion

Exposure to PUA at 15 and 500 nM neither exerted detectable toxic effects on bacterial growth nor functioned as the primary carbon source for the communities studied. Comparative proteomic analyses revealed community-specific reorganization in response to PUA exposure. Native bacterial assemblages, naturally associated with a PUA-producing diatom, displayed stable, regulation-oriented adjustments consistent with physiological accommodation without taxonomic changes. Introduced bacterial assemblages associated with a non-PUA-producing diatom exhibited concentration-dependent responses, with shifting toward maintenance-related processes at higher PUA levels. Our findings demonstrate that PUA-induced effects are characterized by distinct proteomic adjustments conditioned by the ecological history of the community.

## Data Availability

The raw data generated in this study can be found in EBI PRIDE database (www.ebi.ac.uk/pride) under accession PXD075100.

## Footnotes

1
https://www.prophane.de
