# Supplementary material for: Polyunsaturated aldehydes induce distinct proteomic responses in two diatom-associated bacterial communities
Source: Front Microbiol. 2026 Jun 10;17:1838042. doi: 10.3389/fmicb.2026.1838042 (PMC13290949; doi:10.3389/fmicb.2026.1838042)
Supplement: Supplementary file 1 [file Data_Sheet_1.pdf]

Supplementary Material

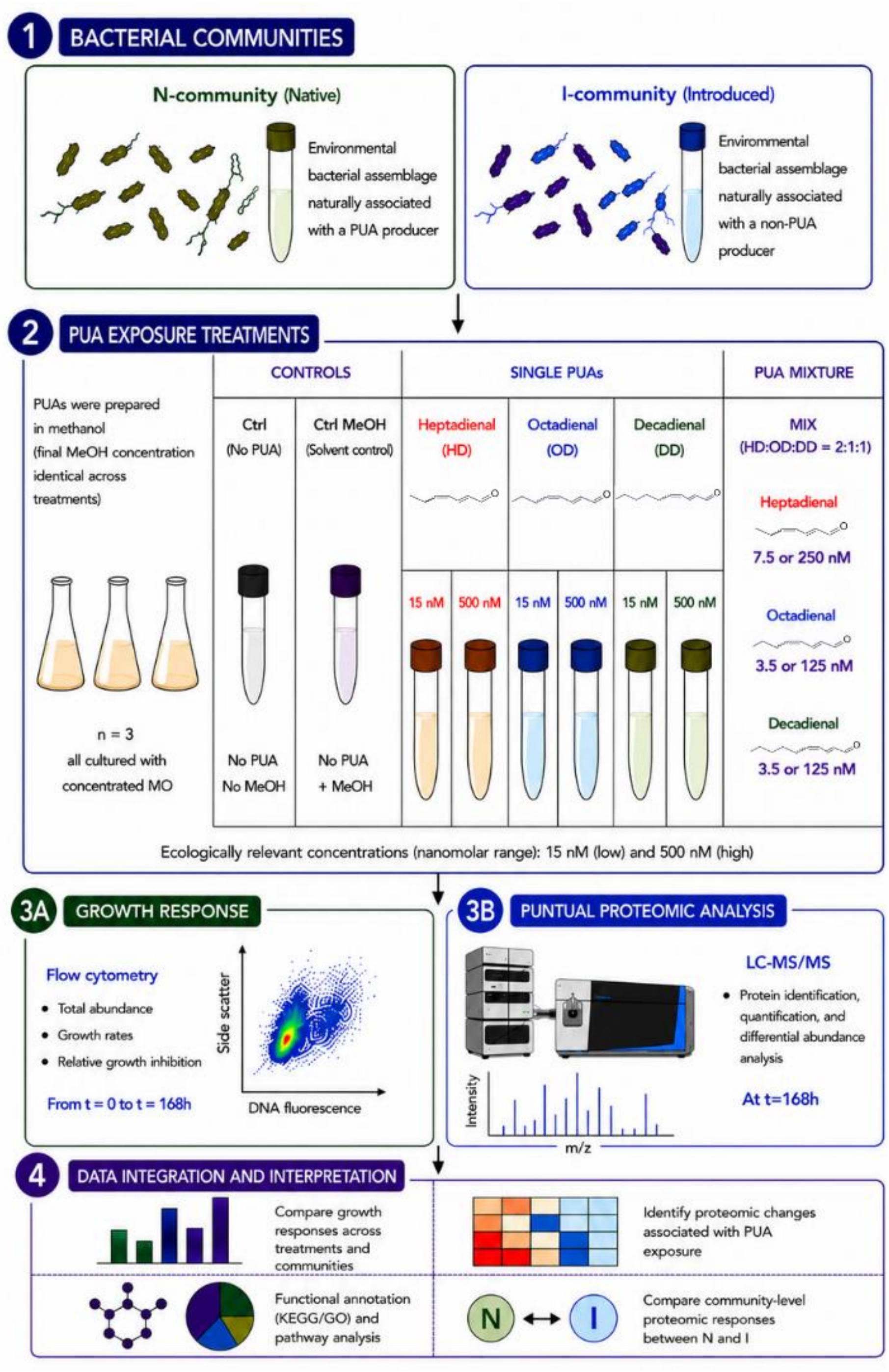

Supplementary Figure S1: Experimental workflow for PUA exposure and subsequent analyses. Two bacterial communities (N-community and I-community) were independently exposed to: control conditions, single PUAs, or a PUA mixture at 15 and 500 nM by triplicate. Cell density was monitored throughout the experiment by flow cytometry. Proteomic characterization was performed at t = 168 h by LC–MS/MS following protein extraction and tryptic digestion, and protein identification and quantification were conducted using a custom database approach. Functional annotation analyses were carried out using OmicsBox, KEGG, GO, and BRITE.

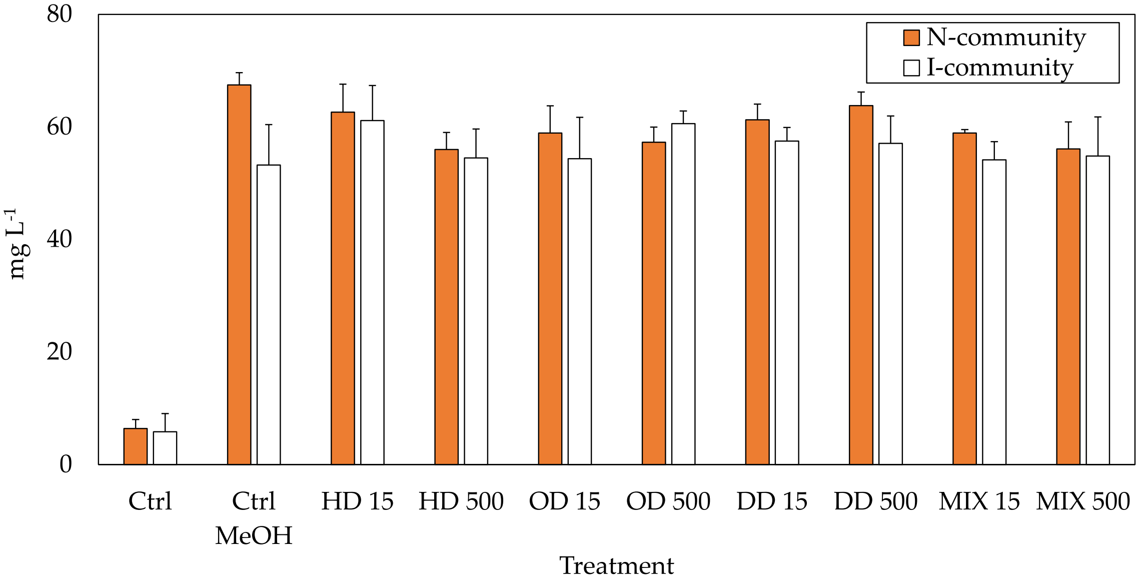

Supplementary Figure S2: Dissolved organic carbon concentration quantified at the end of the experiment (t=168h) in native (N-community) and introduced (I-community) bacterial communities. Data represent means ± standard deviation (n = 3 biological replicates). Treatment key: Ctrl, control cultures; Ctrl MeOH, methanol control; HD, 2*E*,4*E/Z*-heptadienal; OD, 2*E*,4*E/Z*-octadienal; DD, 2*E*,4*E/Z*-decadienal; MIX, mixture of HD, OD, and DD in a 2:1:1 molar proportion.

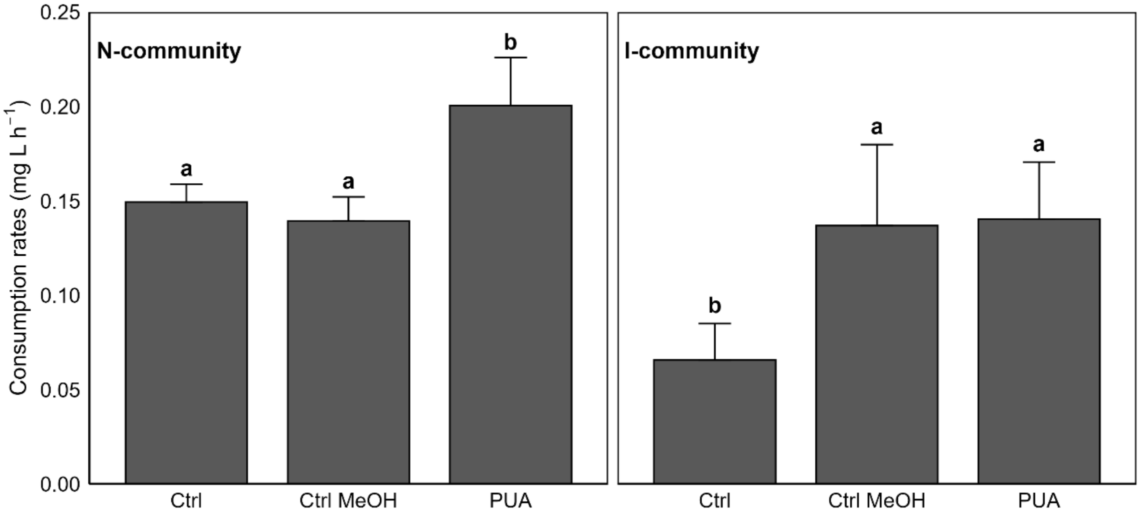

Supplementary Figure S3: Carbon consumption rate quantified in native (N-community) and introduced (I-community) bacterial communities. Data represent means ± standard deviation for Crtl, Crtl MeOH (n = 3) and PUA (n = 24 biological replicates) treatments. Differences among treatments were assessed separately for N- and I-communities using one-way ANOVA, followed by Tukey's HSD post hoc tests. Different letters indicate statistically significant differences among treatments ($p < 0.05$).

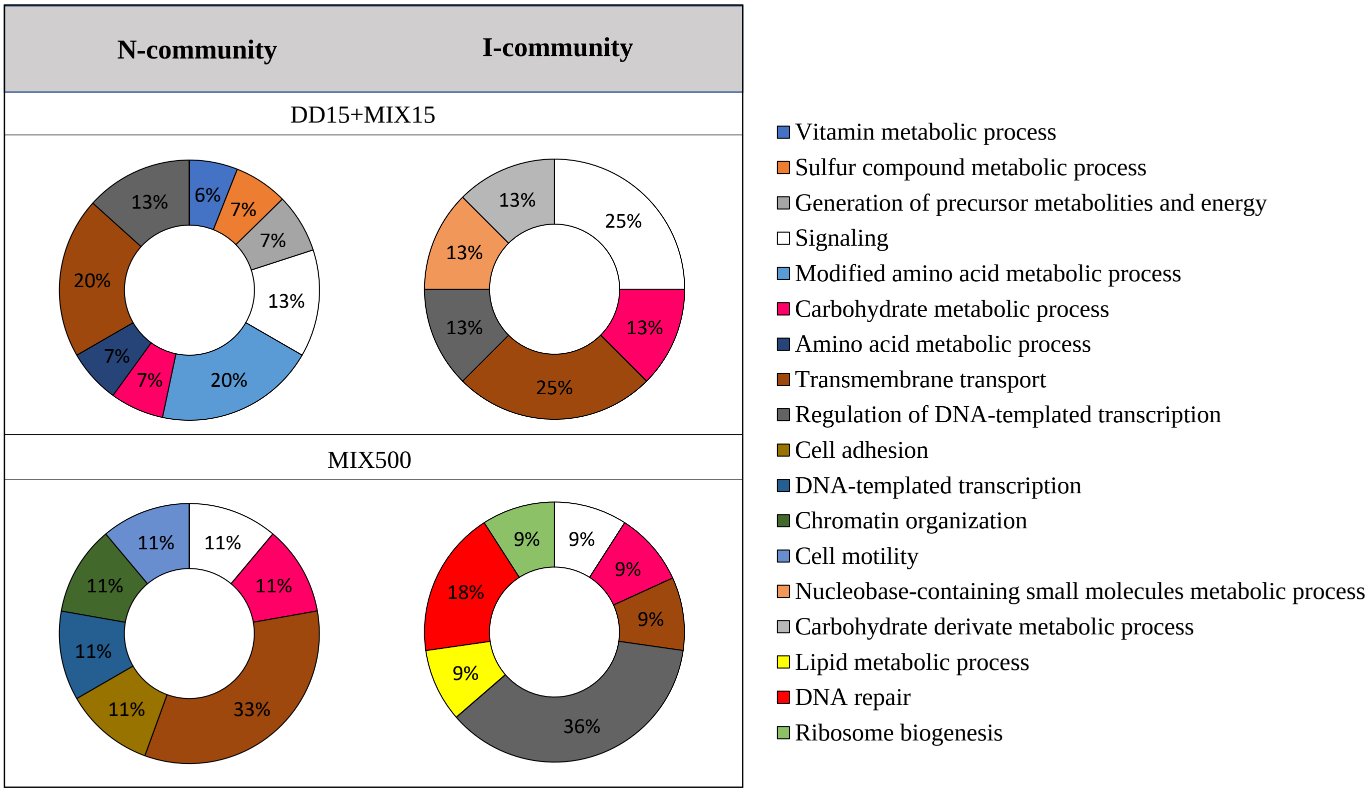

Supplementary Figure S4: GO biological process categories in N- and I-communities under PUA exposure. Gene Ontology (GO) biological process categories identified in the proteomes of the native (N-community) and introduced (I-community) bacterial communities under low (DD15+MIX15) and high (MIX500) PUA exposure conditions. Donut charts show the relative proportion (%) of proteins assigned to each biological process category within each treatment and community.

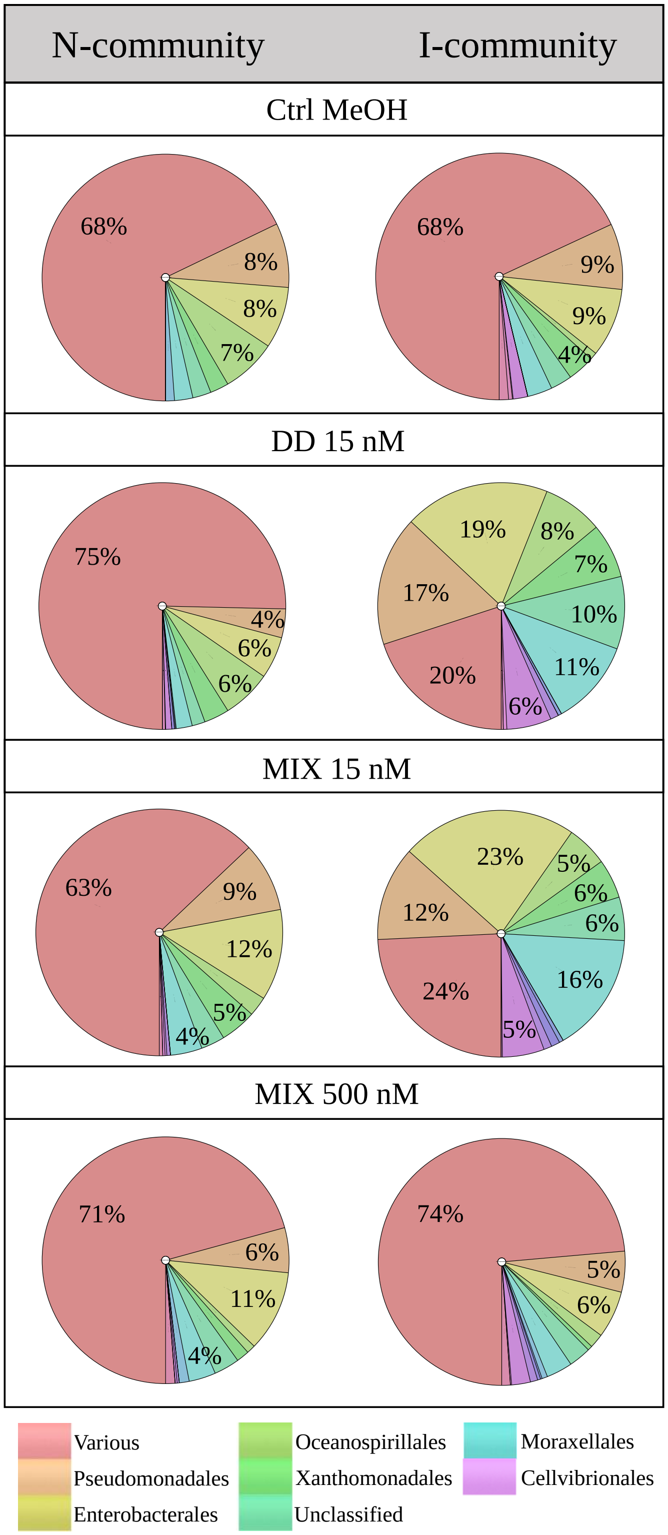

Supplementary Figure S5: KRONA-based taxonomic classification of Gammaproteobacteria-associated proteins under PUA exposure. Pie charts show the relative proportion of proteins normalized abundance assigned to Gammaproteobacteria orders in the proteomic datasets of the native (N-community) and introduced (I-community) bacterial communities under control (Ctrl MeOH), DD 15 nM, MIX 15 nM, and MIX 500 nM treatments.
